# Inverse association of the systemic immune-inflammation index with serum anti-ageing protein Klotho levels in individuals with osteoarthritis: A cross-sectional study

**DOI:** 10.1371/journal.pone.0300674

**Published:** 2024-05-07

**Authors:** Jinlong Zhao, Yinhua Lai, Lingfeng Zeng, Guihong Liang, Xiao Jin, Hetao Huang, Minghui Luo, Jun Liu

**Affiliations:** 1 The Second Clinical College of Guangzhou University of Chinese Medicine, Guangzhou, China; 2 The Second Affiliated Hospital of Guangzhou University of Chinese Medicine (Guangdong Provincial Hospital of Chinese Medicine), Guangzhou, China; 3 The Research Team on Bone and Joint Degeneration and Injury of Guangdong Provincial Academy of Chinese Medical Sciences, Guangzhou, China; 4 The First Affiliated Hospital of Jinan University, Guangzhou, China; 5 Guangdong Second Chinese Medicine Hospital (Guangdong Province Engineering Technology Research Institute of Traditional Chinese Medicine), Guangzhou, China; University of Illinois, UNITED STATES

## Abstract

**Background:**

The association between the systemic immune-inflammation index (SII) and the serum soluble-Klotho concentration (pg/ml) in osteoarthritis (OA) patients is unknown. This study aimed to investigate the relationship between the SII and serum soluble-Klotho levels in OA patients.

**Methods:**

All study data were obtained from the National Health and Nutrition Examination Survey (NHANES) database (n = 1852 OA patients; age range = 40–79 years). The SII and serum Klotho measurement data are from the NHANES mobile examination centre. The SII values were divided into quartiles (Q1-4: 0.02–3.36, 3.36–4.78, 4.79–6.70, and 6.70–41.75). A multivariate linear regression model was constructed to evaluate the association between the SII and serum Klotho levels in OA patients; interaction tests were conducted to test the stability of the statistical results.

**Results:**

Multivariate linear regression revealed a negative linear relationship between the SII and serum Klotho concentration in OA patients (β = -6.05; 95% CI: -9.72, -2.39). Compared to Q1, Q4 was associated with lower serum Klotho concentrations (β = -59.93; 95% CI: -96.57, -23.28). Compared with that of Q1, the β value of Q2-Q4 showed a downwards trend as the SII increased (*P*_trend_ <0.001). The stratified analysis results indicated that the SII had a greater sensitivity in predicting serum Klotho concentrations in OA patients aged 60–79 years (*P*_interaction_ = 0.028).

**Conclusions:**

There was a significant negative linear correlation between the SII and serum Klotho concentration in OA patients. The SII can serve as a predictive indicator of serum Klotho concentrations in OA patients. Klotho may be a potential anti-inflammatory drug for OA treatment.

## Introduction

Osteoarthritis (OA) is the most common chronic degenerative joint disease in middle-aged and elderly people and is characterised by joint pain and dysfunction [1]. Middle-aged and elderly individuals are more prone to OA, and the highest incidence, disability rate, and mortality are observed in OA patients [2,3]. A study [4] based on Global Burden of Disease (GBD) data showed that compared with that of lower back and neck pain, the age-standardised incidence rate (ASIR) of OA increases by 0.32% annually worldwide. OA is closely related to population health, quality of life, the all-cause mortality rate, and medical burden. A large amount of evidence suggests that inflammation plays a central role in the progression of OA [5,6]. The main pathological feature of OA is articular cartilage destruction. With the in-depth study of the relationship between OA and cytokines, inflammatory cytokines such as interleukin-1β (IL-1β) and tumour necrosis factor have been determined to be important factors in joint cartilage destruction [7,8]. IL-1β can upregulate the expression of matrix metalloproteinases (MMPs) and cause degradation of the cartilage matrix [9,10]. Therefore, we believe that there is a close correlation between the overall level of inflammation or local inflammation of the bones and joints and the pathological progression of OA. The systemic immune-inflammation index (SII) is an indicator of neutrophil, lymphocyte, and platelet counts that is used to evaluate inflammation [11–13], and it can more objectively reflect changes in the level of inflammation in the body. The SII can be calculated through routine blood examination, which has the advantages of speed, efficiency, simplicity, and low cost. Previous studies have confirmed that the SII has good clinical value in diagnosing chronic diseases such as tumours, osteoporosis, kidney stones, and rheumatoid arthritis [12–15]. In addition, the SII is also considered to have good application value in predicting serum folate, albumin, vitamin B12, and vascular endothelial growth factor A (VEGF-A) concentrations [16–18].

The Klotho gene (also known as the longevity gene) is related to ageing and is believed to exert antiaging effects through various biological mechanisms [19,20]. With the increase in academic research on the Klotho gene, the function of the Klotho gene has gradually been elucidated. Previous studies have shown that the Klotho gene plays a key biological role in antioxidant, anti-inflammatory, and antiapoptotic mechanisms; kidney protection; and the improvement of calcium and phosphorus metabolism [21–24]. The SII is considered a composite indicator reflecting systemic inflammation, with stronger specificity for inflammation indicators than for single inflammatory markers such as neutrophils and white blood cells [12,13]. Wu et al.’s study showed that Klotho levels were significantly correlated with inflammatory markers such as uric acid, C-reactive protein, white blood cell count, and mean platelet volume [25]. Their research conclusion suggested that Klotho is involved in the inflammatory process and plays a protective role, which means that Klotho can serve as a reverse indicator of inflammation [25]. The Klotho gene has three subtypes [26]: α-Klotho, β-Klotho, and γ-Klotho. Currently, the most researched and relatively understood subtype is α-Klotho, which is expressed mainly in proximal renal tubular epithelial cells [27,28]. The soluble Klotho protein plays an important regulatory role in oxidative stress and can inhibit age-related inflammatory responses and endoplasmic reticulum stress [29]. OA is a degenerative joint disease closely related to ageing and inflammation. Based on the close relationship between the Klotho gene and ageing and inflammation, we believe that the overall degree of inflammation in OA patients may affect serum Klotho concentrations.

However, the association between the SII and serum Klotho has not yet been revealed. To fill this gap, we used data from the National Health and Nutrition Examination Survey (NHANES) database in the United States to explore the relationship between the SII and serum Klotho concentrations in OA patients. Our expectation was that this study would elucidate the relationship between the SII and serum Klotho concentration in patients with OA and provide a new perspective for further understanding the role of serum Klotho levels in the pathological progression of OA.

## Materials and methods

### Data sources and participants

The raw data that we used were taken from the NHANES database. The NHANES is a cross-sectional survey conducted by the Centers for Disease Control and Prevention (CDC) in the United States [30] aimed at collecting health and nutritional information for the general population in the United States. The survey subjects of the NHANES were selected through stratified cluster multistage sampling, which can serve as a representative sample of U.S. residents. Due to the independent variables and endpoint outcomes of this study being the SII and serum Klotho concentrations, we selected only five survey periods from 2007 to 2016 with sufficient measurement data to calculate these two variables. All participants signed an informed consent form [31]. The NHANES was approved by the Ethics Review Committee of the National Center for Health Statistics in the United States [31].

**Fig 1:**
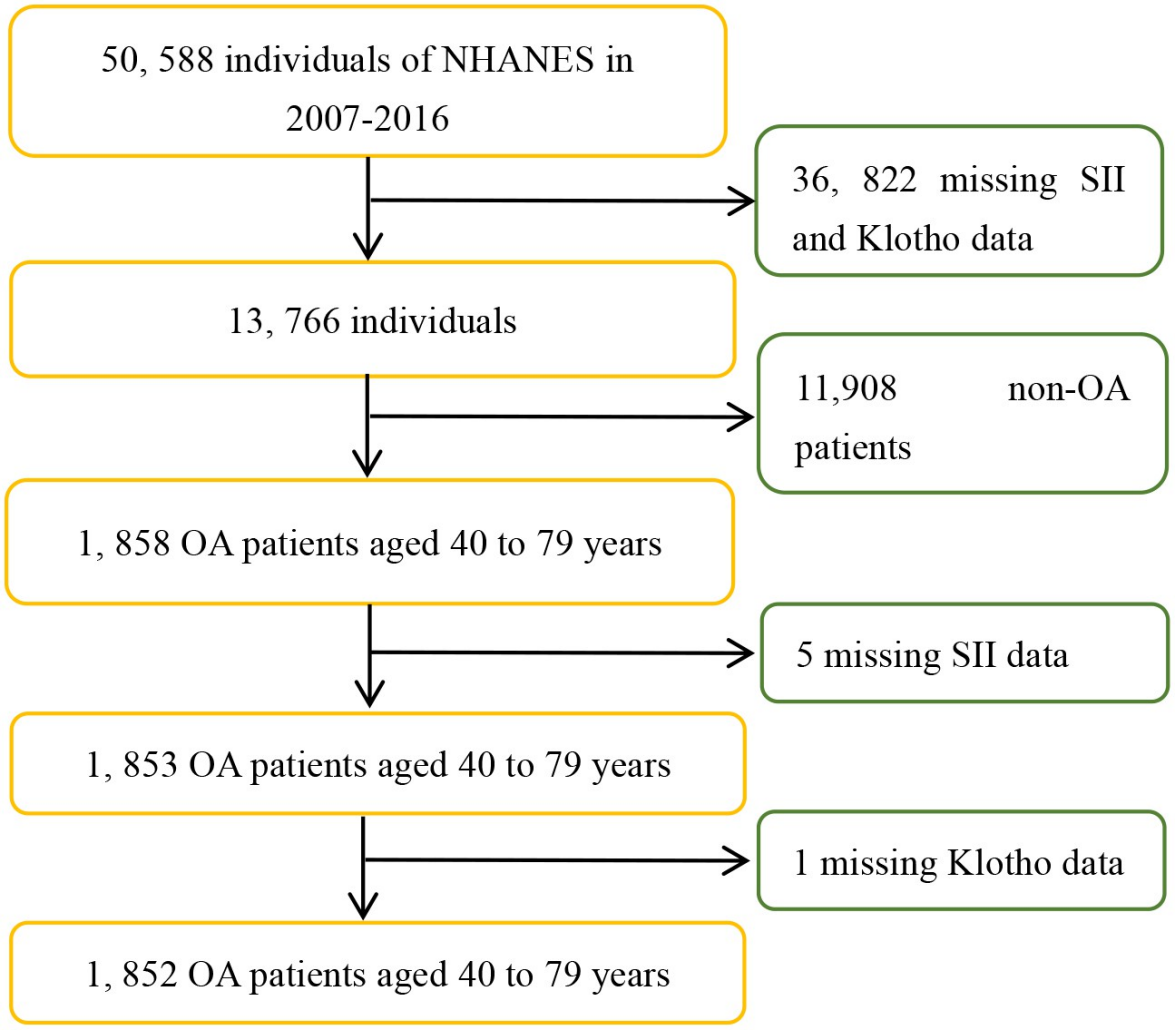
Flow chart of study patients.

A total of 50588 participants were included in the survey from 2007 to 2016. The self-reported health status data of a total of 1858 OA patients were obtained from personal interviews, and their diagnoses were mainly based on their reports of previous diagnoses of OA by doctors. In summary, the inclusion and exclusion criteria were as follows: (1) Participants with complete data on serum Klotho levels and the SII were included, while participants missing this information were excluded. (2) Specifically, participants with only missing covariate data were not excluded; those missing values were set as dummy variables in the statistical analysis. (3) Apart from this, there were no other exclusion or inclusion criteria. Ultimately, a total of 1852 OA patients (range: 40 to 79 years old) were included in this study. The inclusion and exclusion process of the research subjects is shown in Fig 1.

### Measurement of the SII

The SII is an immune-inflammation indicator calculated based on the counts of neutrophils, platelets, and lymphocytes in peripheral blood. The calculation formula was as follows: SII = platelet count × neutrophil count/lymphocyte count [11]. Blood samples from all participants were collected at the NHANES mobile examination centre (MEC). The instruments used for whole blood cell count analysis were a Coulter HMX (Coulter Electronics Ltd., Bedfordshire, UK) [32] or Beckman Coulter DXH 800 (Beckman Coulter, Brea, CA, USA) [33]. A detailed description of the laboratory method for whole blood cell counting can be found on the NHANES website.

### Serum soluble-Klotho concentrations

We collected serum soluble-Klotho (pg/ml) data measured by the NHANES among participants from 2007 to 2016. Whole blood samples were obtained from participants from 2007 to 2016, and serum was prepared by centrifugation at the MEC. The serum samples were stored at -80°C by the CDC in Atlanta, Georgia (GA). Serum Klotho concentrations were measured using a commercially available ELISA kit (IBL International, Japan) from a CDC-certified laboratory [34]. The Klotho concentrations of all serum samples were analysed twice, and the average of these two measured concentrations was used as the final measurement value. In addition, the sensitivity of the Klotho concentration measurement was 6.15 pg/mL. The sample information, experimental measurement details, and quality control of the Klotho concentration measurements can be found on the official NHANES website [35].

### Assessment of covariates

The selection of covariates in this study was mainly based on the literature and the variables recognised by the academic community [36–40], which primarily included sociodemographic factors. The covariates mainly included age (years), sex, race (non-Hispanic white, non-Hispanic black, American Mexican, and others), body mass index (BMI), smoking status, and alcohol consumption. We divided the patients into three groups based on BMI values: <25.0, 25.0–29.9, and ≥30 kg/m^2^. We determined the smoking status of participants based on their questionnaire answers for the number of cigarettes smoked and the smoking frequency. Participants who smoked fewer than 100 cigarettes in their lifetime were considered nonsmokers (never). If a participant smoked more than 100 cigarettes in their lifetime but did not smoke at the time of the study, they were considered to have previously smoked (former). If a participant smoked more than 100 cigarettes in their lifetime and smoked daily or occasionally at the time of the study, they were considered current smokers (current). According to Rattan et al.’s classification criteria [41], we classified alcohol consumption levels into never, former, low, moderate, and heavy. In addition, we extracted six diseases, namely, diabetes [42], chronic kidney disease (CKD) [43], hypertension [44], hyperlipidemia [45], chronic obstructive pulmonary disease (COPD) [46], and coronary heart disease (CHD) [47], as covariates. The diagnosis of these six diseases was based on clear diagnostic criteria [42–47] and matched with measurement indicators in the NHANES database; each participant included in the study voluntarily reported taking disease-related drugs, such as hypoglycaemic drugs and antihypertensive drugs; or participants reported having been diagnosed by a doctor.

### Statistical analyses

The data analysis in this study was carried out according to the CDC’s statistical guidelines [48]. All data extraction and analysis were conducted using R software (version 4.2.2, http://www.R-project.org, The R Foundation) and EmpowerStats software (version 4.1, www.empowerstats.com, X&Y solutions, Inc. Boston, MA, USA). All tests were two-tailed, and α was set to 0.05. Continuous variables are represented by weighted means and standard deviations (SDs); categorical variables are expressed as frequencies and weighted percentages. We used a linear regression model to evaluate the relationship between the SII and serum Klotho concentration. Considering that the absolute value of the SII is relatively large and to enhance the statistical efficiency of the data and facilitate clinical application, we divided all the SII values by 100 as the raw data. Therefore, in this study, an increase of one unit in the SII means an increase of 100 in the absolute value of the SII. To test the stability of the influence of the SII on the Klotho level in different regression models, we divided the SII values into four quartiles. The quartile ranges of the SII were 0.02–3.36 (Q1), 3.36–4.78 (Q2), 4.79–6.70 (Q3) and 6.70–41.75 (Q4). The SII was entered into the model as a continuous variable and as a categorical variable (quartile grouping). To further test the stability of the relationship between the SII and Klotho, we constructed three regression models to gradually adjust for confounding factors: Model 1 did not adjust for covariates; Model 2 adjusted for age, sex, and race; and Model 3 further adjusted for BMI, smoking status, alcohol consumption, diabetes, CKD, hypertension, COPD, CHD, and hyperlipidemia on the basis of Model 2. We further conducted interaction tests (subgroup analysis) on these covariates to comprehensively evaluate the reliability of our research conclusions.

## Results

### Baseline characteristics of study individuals

**Fig 2:**
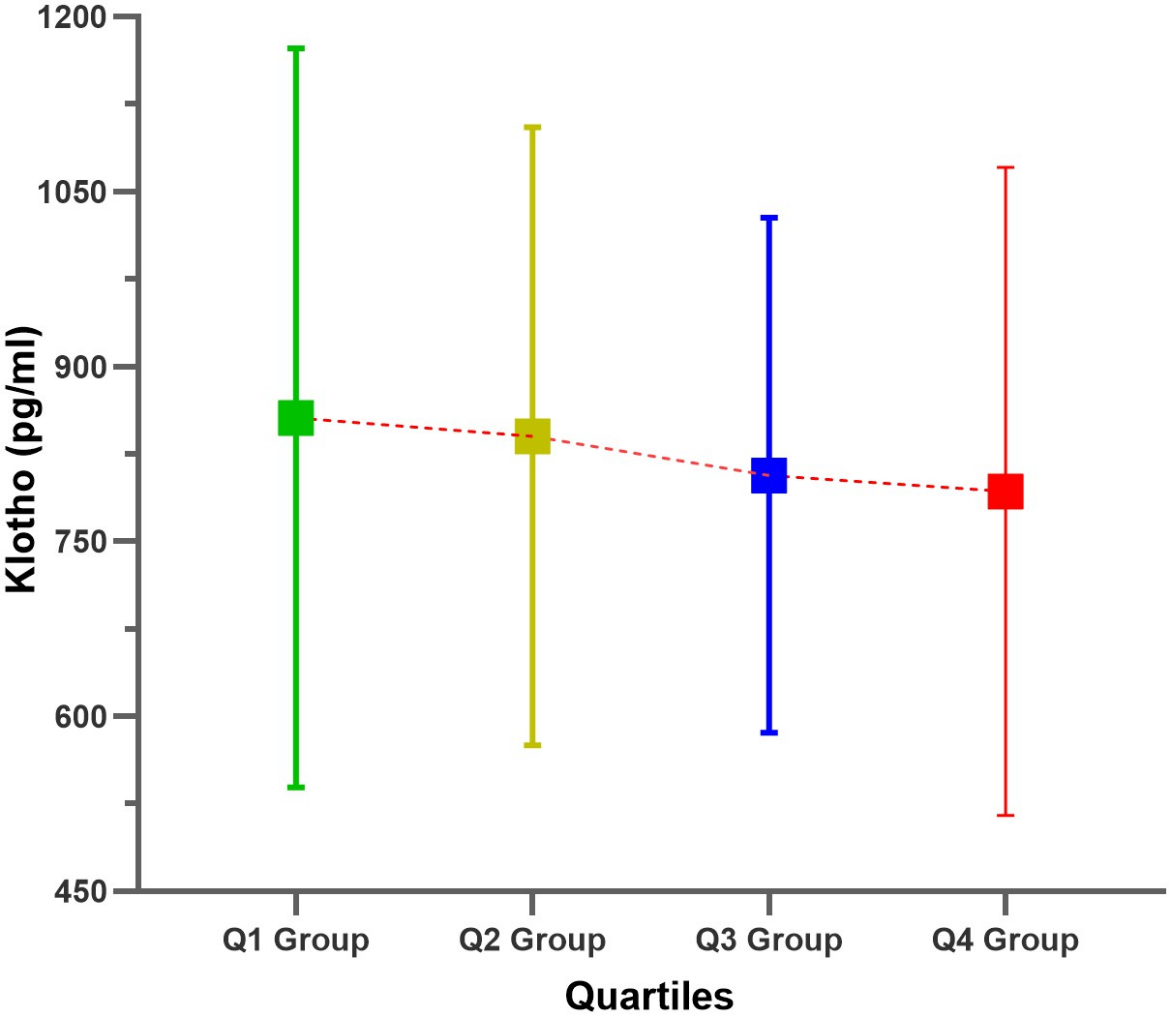
Relationships between the SII quartiles and serum Klotho levels in patients with OA.

A total of 1852 OA patients aged 40–79 years were included in this study. The weighted average serum Klotho concentrations in the SII Q1-Q4 groups were 855.81, 840.22, 806.68, and 792.87, respectively, and the differences between the groups were statistically significant (*P* = 0.002). In addition, as the quartile of the SII increased, the serum Klotho levels of OA patients tended to decrease (Fig 2). In general, the SII quartiles were significantly different in terms of race/ethnicity, alcohol consumption, CKD status, neutrophil count, lymphocyte count, and platelet count. The basic characteristics of the study population grouped according to the SII quartiles are shown in Table 1.

**Table 1:** Characteristics of individuals by SII quartile from NHANES, 2007–2016.

Characteristic	SII levels (N = 1852)				
	Q1 (<3.36)	Q2 (3.36–4.78)	Q3 (4.79–6.70)	Q4 (> 6.70)	*P-*Value
Age, years					0.133
40–59	143 (36.74)	165 (42.23)	151 (37.96)	158 (39.78)	
60–69	189 (40.42)	168 (34.94)	179 (39.81)	153 (33.11)	
70–79	131 (22.84)	130 (22.82)	133 (22.23)	152 (27.11)	
Sex					0.13
Male	175 (36.74)	170 (39.99)	170 (36.39)	163 (32.72)	
Female	288 (63.26)	293 (60.01)	293 (63.61)	300 (67.28)	
Race/ethnicity					0.0001
Non-Hispanic White	218 (76.43)	286 (84.74)	290 (85.66)	315 (86.96)	
Non-Hispanic Black	121 (11.60)	72 (5.45)	72 (4.02)	48 (3.87)	
American Mexican	40 (3.08)	46 (2.91)	46 (3.44)	44 (2.81)	
Others	84 (8.89)	59 (6.91)	59 (6.89)	56 (6.36)	
BMI (kg/m^2^)					0.765
<25	76 (18.57)	80 (19.14)	88 (20.66)	83 (19.22)	
25.0–29.9	152 (34.22)	132 (29.34)	124 (29.66)	128 (28.56)	
≥ 30	228 (46.70)	245 (50.29)	243 (48.61)	244 (50.90)	
Missing	7 (0.51)	6 (1.23)	8 (1.07)	8 (1.32)	
Smoking status					0.094
Never	234 (49.51)	220 (44.04)	219 (48.06)	187 (43.76)	
Current	73 (14.70)	72 (15.03)	70 (13.64)	95 (19.05)	
Former	156 (35.79)	170 (40.48)	174 (38.30)	181 (37.19)	
Missing	0	1 (0.45)	0	0	
Alcohol consumption					0.003
Never	73 (10.24)	53 (8.79)	56 (8.70)	58 (10.15)	
Former	97 (15.44)	99 (17.12)	108 (20.04)	126 (24.57)	
Low to moderate	221 (56.42)	241 (57.88)	241 (60.09)	222 (54.00)	
Heavy	53 (13.65)	46 (10.12)	37 (8.25)	32 (7.29)	
Missing	19 (4.25)	24 (6.09)	21 (2.91)	25 (3.99)	
Diabetes					0.456
No	328 (79.71)	341 (76.27)	331 (76.83)	316 (75.22)	
Yes	125 (20.29)	122 (23.73)	132 (23.17)	147 (24.78)	
CKD					0.001
No	368 (84.79)	356 (77.26)	338 (76.72)	315 (74.31)	
Yes	95 (15.21)	104 (21.96)	119 (21.21)	139 (24.56)	
Missing	0	3 (0.78)	6 (2.07)	9 (1.13)	
Hypertension					0.263
No	169 (42.24)	167 (39.97)	157 (38.68)	134 (35.79)	
Yes	294 (57.76)	296 (60.03)	306 (61.32)	329 (64.21)	
COPD					0.244
No	424 (89.83)	421 (91.04)	414 (89.62)	397 (87.12)	
Yes	39 (10.17)	42 (8.96)	49 (10.38)	66 (12.88)	
CHD					0.884
No	433 (93.88)	435 (94.56)	429 (94.16)	425 (93.06)	
Yes	28 (6.01)	27 (5.16)	33 (5.76)	37 (6.83)	
Missing	2 (0.10)	1 (0.28)	1 (0.05)	1 (0.11)	
Hyperlipidemia					0.765
No	75 (15.07)	84 (17.46)	68 (15.50)	69 (16.13)	
Yes	388 (84.93)	379 (82.54)	395 (84.50)	394 (83.87)	
Klotho (pg/ml)	855.81±316.96	840.22±265.11	806.68±220.88	792.87±277.93	0.002
Neutrophils count (10^3^/μL)	2.92±0.90	3.91±0.99	4.48±1.17	5.83±1.95	<0.001
Lymphocyte count (10^3^/μL)	2.36±1.41	2.14±0.60	1.93±0.63	1.77±0.64	<0.001
Platelet count (10^3^/μL)	201.79±47.37	223.96±45.92	241.25±53.97	285.75±69.28	<0.001

N: Number of observed; SII: Systemic immune-inflammation index; BMI: Body mass index; CKD: Chronic kidney disease; COPD: Chronic obstructive pulmonary disease; CHD: Coronary heart disease.

For continuous variables: Mean +/- SD; *P*-value was calculated by weighted linear regression model.

For categorical variables: Unweighted N and weighted %; *P*-value was calculated by weighted chi-square test.

### Association between the SII and Klotho

All covariate-adjusted models (Model 3) showed a significant association between a higher SII and lower serum Klotho concentrations (β = -6.05, 95% CI: -9.72 to -2.39). The Model 3 results showed that compared to those in the Q1 group, the serum Klotho concentrations in the Q4 group were significantly lower (β = -59.93, 95% CI: -96.57 to -23.28) (*P* = 0.0013). Compared with that of the Q1 group, the β value of the Q2 to Q4 groups tended to decrease with increasing SII (*P*_trend_ < 0.001). The results of univariate and multivariate linear regression analyses (Table 2) indicated that higher SII values are associated with lower serum Klotho concentrations, indicating a negative linear relationship between the two measures.

**Table 2:** Association of SII with Klotho (pg/ml) in 1,852 participants aged 40 to 79 years.

Exposure	Model 1	*P*-value	Model 2	*P*-value	Model 3	*P*-value
	β (95% CI)		β (95% CI)		β (95% CI)	
SII	-6.41 (-10.02, -2.80)	0.0005	-6.25 (-9.89, -2.62)	0.0008	-6.05 (-9.72, -2.39)	0.001
SII quartile						
Q1	Reference		Reference		Reference	
Q2	-15.60 (-50.98, 19.78)	0.386	-14.20 (-49.83, 21.42)	0.435	-13.77 (-49.26, 21.72)	0.447
Q3	-49.13 (-85.46, -12.80)	0.008	-48.74 (-85.32, -12.15)	0.009	-46.49 (-82.96, -10.03)	0.0125
Q4	-62.95 (-99.2, -26.69)	0.0006	-61.56 (-98.18, -24.93)	0.001	-59.93 (-96.57, -23.28)	0.0013
*P* for trend	<0.001		<0.001		<0.001	

Model 1: No covariates were adjusted.

Model 2: Age, sex, and race/ethnicity were adjusted.

Model 3: Age, sex, and race/ethnicity, BMI, smoking status, alcohol consumption, diabetes, CKD, hypertension, COPD, CHD, and hyperlipidemia were adjusted.

### Subgroup analysis

To further test the reliability of the regression model, we conducted a subgroup analysis on all the covariates (Table 3). We found a significant negative correlation between the SII and Klotho concentration, which was not affected by sex, race/ethnicity, BMI, smoking status, alcohol consumption, diabetes, CKD, hypertension, COPD, CHD, or hyperlipidemia (all *P values* for interactions ≥ 0.05). In the 40–59, 60–69, and 70–79 age groups, the β values were 0.02, -11.32, and -7.28, respectively, and there was an interaction effect between the groups (*P* for interaction = 0.028), indicating a significant negative correlation between the SII and the serum Klotho concentration in the 60–79 age group compared to that in the 40–59 age group.

**Table 3:** Subgroup analysis.

Parameters	N	β (95% CI)	*P* for Interaction
Age, years			0.028
40–59	617	0.02 (-5.97, 6.01)	
60–69	689	-11.32 (-17.40, -5.25)	
70–79	546	-7.28 (-14.21, -0.34)	
Sex			0.740
Male	678	-4.72 (-11.35, 1.92)	
Female	1174	-6.04 (-10.40, -1.68)	
Race/ethnicity			0.754
Non-Hispanic White	1109	-5.97 (-9.95, -2.00)	
Non-Hispanic Black	294	-14.74 (-34.24, 4.76)	
Non-Hispanic Asian	180	-9.72 (-27.06, -7.62)	
BMI			0.05
<25	327	-6.00 (-16.11, 4.11)	
25.0–29.9	536	-13.47 (-20.68, -6.26)	
≥ 30	960	-1.29 (-6.16, 3.59)	
Smoking status			0.573
Never	860	-5.80 (-11.99, 0.39)	
Current	310	-2.59 (-9.30, 4.13)	
Former	681	-7.42 (-13.70, -1.13)	
Alcohol consumption			0.259
Never	240	-11.90 (-24.15, 0.35)	
Former	430	-8.49 (-15.12, -1.87)	
Low to moderate	925	-2.03 (-7.19, 3.13)	
Heavy	168	-12.21 (-29.29, 4.87)	
Diabetes			0.124
No	1326	-7.96 (-12.33, -3.69)	
Yes	526	-1.39 (-8.71, 5.93)	
CKD			0.926
No	1377	-5.83 (-10.19, -1.46)	
Yes	457	-6.02 (-13.29, 1.25)	
Hypertension			0.194
No	627	-8.72 (-14.19, -3.26)	
Yes	1255	-3.93 (-8.81, 0.94)	
COPD			0.075
No	1656	-4.81 (-8.97, -0.65)	
Yes	196	-12.54 (-20.07, -5.01)	
CHD			0.34
No	1722	-6.50 (-10.45, -2.55)	
Yes	125	-0.51 (-12.35, 11.33)	
Hyperlipidemia			0.05
No	296	-14.26 (-23.07, -5.46)	
Yes	1556	-4.39 (-8.39, -0.39)	

N: Number of observed.

Age, sex, and race/ethnicity, BMI, smoking status, alcohol consumption, diabetes, CKD, hypertension, COPD, CHD, and hyperlipidemia were all adjusted except the variable itself.

## Discussion

The Klotho gene is an antiaging gene that has anti-inflammatory, antioxidant, and regulatory effects on calcium and phosphorus metabolism [19,24]. OA patients generally experience low-grade inflammatory reactions and exhibit a chronic inflammatory state with age [49]. The SII is a simple and easily accessible indicator for evaluating overall inflammatory status and has been widely used to evaluate changes in inflammatory levels in postoperative or tumour populations. In this study, we explored the correlation between the SII and serum Klotho concentration in OA patients. To our knowledge, this is the first study to explore this topic. In this study, the relationship between the SII and Klotho concentration, whether correlational or causal, warrants attention. The results of this study indicate a negative linear relationship between the SII and serum Klotho concentration in OA patients. This finding supports the use of the SII as a predictive indicator of Klotho concentration, and Klotho may serve as a potential therapeutic drug for OA. In OA patients, if there is a causal relationship between Klotho and the SII, promoting the secretion of endogenous Klotho protein or the administration of exogenous Klotho protein will provide significant clinical value in the treatment of OA. Moreover, research on the use of Klotho as a therapeutic drug for OA may greatly promote the progression of OA treatment.

Joint cartilage injury and inflammatory changes are the main pathological features in OA patients. Animal experimental studies have shown that high expression of Klotho can reduce articular cartilage degradation [50], and this study also suggested that Klotho is an antagonist of endogenous Wnt/β-catenin activity. In addition, this study confirmed that low expression of Klotho in OA cartilage can activate Wnt/β-catenin signalling and induce cartilage damage [50]. The expression level of Klotho in the articular cartilage of OA mice was significantly reduced [51]. High expression of Klotho inhibits chondrocyte apoptosis through the thioredoxin/peroxiredoxin (Trx/Prx) family and the ROS/TXNIP/NLRP3 signalling pathway [51], indicating that higher concentrations of serum Klotho can significantly delay the pathological progression of OA. Klotho is believed to have strong antioxidant and anti-inflammatory effects. One study [52] suggested that targeted regulation of Klotho protein can affect the expression of inflammatory factors, such as FGF23, Bcl-2, Bax, TGF-β1, Caspase 3 and Caspase 8, and thus affect the pathological progression of OA. Li et al. reported that a decrease in Klotho expression in macrophages and peripheral blood monocytes leads to an increase in the expression of the inflammatory mediators MMP-9, IL-6, and TNF-α [53]. Previous studies have confirmed that recombinant Klotho protein plays an important therapeutic role in reducing aseptic inflammation [54]. An increase in the SII reflects an increase in neutrophil and platelet counts, as well as an increase in the levels of various cytokines (and/or a decrease in lymphocyte counts) [55,56]. A reduction in lymphocytes during inflammation can increase the production of proinflammatory cytokines, induce oxidative stress, and promote cell apoptosis [55,56], thereby exacerbating the inflammatory response and promoting disease progression. Given the important biological role of Klotho protein in the progression of OA and the anti-inflammatory and antioxidant functions of Klotho, we believe that the SII may be an effective indicator to assist in evaluating serum Klotho protein concentrations in OA patients. In addition, the use of Klotho as a drug for treating OA may be an important topic for future drug development.

To further test the stability of this regression model, we conducted stratified analyses on all covariates. Notably, the negative linear association between the SII and serum Klotho concentration in OA patients was not affected by sex, race/ethnicity, BMI, smoking status, alcohol consumption, diabetes, CKD, hypertension, COPD, CHD, or hyperlipidemia, indicating that the conclusions of this study are relatively stable. Compared with the 40–59-year-old population, there seems to be a more significant negative association between the SII and serum Klotho concentrations in the 60–79-year-old population, indicating that changes in inflammation levels in older OA patients are more likely to affect Klotho expression. Ageing is closely related to the level of chronic inflammation throughout the body. As age increases, the expression of the Klotho gene decreases [57]. Klotho gene-deficient mice age 3–4 weeks after birth [58], while Klotho overexpression prolongs the survival time of mice. OA is an age-related degenerative joint disease, and there is a positive correlation between age and OA-related inflammation [59,60]. We believe that the SII has greater sensitivity for evaluating serum Klotho concentrations in OA patients older than 60 years. Carreras-Badosa et al. reported that Klotho was a protective factor against visceral fat accumulation in people with high body weight [61], but the relationship between Klotho and the SII (inflammatory level) still needs to be further studied. Similarly, a high Klotho concentration has been confirmed to be associated with a reduced risk of hyperlipidemia [62], but its mechanism remains to be further explored. In our study, we found that there was a slight negative correlation between the SII and serum Klotho levels in different BMI groups and hyperlipidemia in OA patients, but more studies are needed in the future to provide stronger epidemiological evidence.

## Limitations

This study has several limitations that should be appropriately considered when applying the conclusions of this study. This was a cross-sectional study, which means that causal relationships could not be determined. Therefore, the determination of causality still needs to be confirmed by further prospective cohort studies or Mendelian randomised studies in the future. Second, due to the lack of measurement data for the SII or serum Klotho protein in some participants, we had to exclude them from this study. Due to the lack of these samples, there may be bias in the conclusions of this study. Finally, we need to note that the target population of this study is based on the population of the United States, which may make the conclusions of this study unsuitable for extrapolation to populations in other countries or continents.

## Conclusions

This study revealed a significant negative linear relationship between the SII and serum Klotho concentration in OA patients, indicating that a higher SII is associated with lower Klotho concentration. The SII can serve as a predictive indicator of serum Klotho concentrations in OA patients, and Klotho may serve as a potential anti-inflammatory drug for OA treatment. The causal relationship between the SII and serum Klotho concentration still needs further prospective cohort studies or Mendelian randomised studies for verification.

## Supporting information

S1 Data Supporting information caption for file "original data.xlsx": Original data used for this study. (XLSX)
